# First Brazilian pediatric hospital to adopt 1-hour preoperative fasting time for clear fluids for elective surgeries

**DOI:** 10.6061/clinics/2021/e3336

**Published:** 2021-09-20

**Authors:** Priscilla Ferreira Neto Cardoso, Vinicius Caldeira Quintão, Bruno Perini, Maria José Carvalho Carmona, Ricardo Vieira Carlos, Cláudia Marquez Simões

**Affiliations:** I Disciplina de Anestesiologia, Faculdade de Medicina FMUSP, Universidade de Sao Paulo, Sao Paulo, SP, BR.; II Instituto da Crianca e do Adolescente, Hospital das Clinicas HCFMUSP, Faculdade de Medicina, Universidade de Sao Paulo, Sao Paulo, SP, BR.; III Instituto do Cancer do Estado de Sao Paulo, Hospital das Clinicas HCFMUSP, Faculdade de Medicina, Universidade de Sao Paulo, Sao Paulo, SP, BR.; IV Servicos Medicos de Anestesia (SMA), Hospital Sirio-Libanes, Sao Paulo, SP, BR.

Dear Editor,

There is a worldwide trend of reduced preoperative fasting time in children. The European Society of Anaesthesiology and Intensive Care guidelines published in 2011 and the American Society of Anesthesiologists guidelines, published in 2011 and reviewed in 2017, recommended a preoperative fasting time of 2 hours for clear liquids. In 2018 (1), a consensus to reduce the preoperative fasting time to 1 hour for clear fluids was published by the Association of Paediatric Anaesthetists of Great Britain and Ireland, the European Society for Paediatric Anaesthesiology, and L'Association des Anesthésistes-Réanimateurs Pédiatriques d'Expression Française. Similarly, other societies, such as the Canadian Pediatric Anesthesia Society and the Society for Paediatric Anaesthesia in New Zealand and Australia, have adopted the 1-hour fasting time for clear liquids, endorsing the consensus of the European pediatric anesthesia societies. In 2019 (2), this topic was raised by Brazilian authors, and the Anesthesia Division of the Instituto da Criança e do Adolescente, Hospital das Clínicas, Faculdade de Medicina, Universidade de São Paulo was the first to adopt the reduced fasting time in Brazil.

A critical question confronting anesthesiologists is patient safety, since inadequate fasting times can lead to gastric content aspiration. The APRICOT study (3) revealed a gastric content aspiration risk of 9.3/10,000, and none of the children had severe complications. Are only solid foods the cause of gastric content aspiration? Although we cannot affirm this, the aspiration of clear fluids does not seem to occur in such a short time of 1 hour, or cause serious adverse events when the amount of clear fluid consumed is within limits. Thomas et al. (1) stated that consuming 3 mL/kg of clear liquids is a safe volume for preoperative fasting of 1 hour for children aged over 1 year. This is not a new concept. In fact, Lister (4) affirmed in 1883 that tea consumed 2 hours before anesthesia could be beneficial.

However, is this reduction in the preoperative fasting time for clear liquids from 2 hours to 1 hour significant? How would this contribute in improving the quality of patient care in the preoperative period? Considering this question, some quality improvement projects have already been published (5,6). They contributed to behavioral changes that involved either the hospital's multidisciplinary team or the parents and children. It is known that the fasting period is often much longer than the recommended 1 or 2 hours. This protocol depends on cultural issues, because for many years, the “nil per os,” *i.e.*, nothing consumed through the mouth after midnight, was the standard. It was believed that with prolonged fasting time, the risk of gastric content aspiration decreases. This adverse event of gastric content aspiration is most feared by anesthesiologists and is responsible for the long fasting times recommended. There are only a few studies on preoperative fasting in pediatric patients. An additional challenge is that children usually do not readily consume different drinks such as tea, coconut water, or maltodextrin-containing solutions, making compliance difficult.

Our institution, which is the first to adopt the 1-hour preoperative fasting time for clear fluids in children, has a task force that involves pediatric anesthesiologists, pediatric surgeons, pediatricians, a nutrition team, and a nursing team. Everyone acts in coordination, following the same recommendations. With this pioneering quality improvement project in Brazil to reduce the preoperative fasting time for clear liquids, we hope to initiate a change in the national scenario to improve the quality of Brazilian pediatric anesthesia, as has been observed worldwide.
